# Diet, Gastric Microbiota, and Metabolites in Gastric Tumorigenesis

**DOI:** 10.34133/research.0693

**Published:** 2025-05-12

**Authors:** Lanping Jiang, Harry Cheuk-Hay Lau, Ruijie Zeng, Jun Yu

**Affiliations:** Institute of Digestive Disease, Department of Medicine and Therapeutics, State Key Laboratory of Digestive Disease, Li Ka Shing Institute of Health Sciences, The Chinese University of Hong Kong, Hong Kong SAR, China.

## Abstract

Gastric cancer (GC) is one of the most common cancers worldwide particularly in Asian populations, and certain diets have been associated with increased risk of GC. Recent advances in microbial profiling technology have facilitated investigations on microbes residing on the gastric mucosa and increasing evidence has revealed the critical roles of non-*Helicobacter pylori* gastric microbes in gastric tumorigenesis. On the other hand, diets can affect microbial communities, causing compositional and functional shift of the microbiota. In this review, we summarize the influence of various diets including processed meat, salt-preserved food, high-fat diet, and alcohol on the development and progression of GC. We also explore microbial metabolites and host–microbe interactions in gastric tumorigenesis, alongside dietary interventions targeting the microbiota for the prevention and management against GC.

## Introduction

Gastric cancer (GC) ranks as the fifth most common cancer and the third leading cause of cancer-related deaths worldwide. It is estimated that there are over 1 million new cases of GC each year, resulting in more than 784,000 deaths globally [1]. Despite advancements in treatment options, the rising of the aging population is projected to drive higher GC incidence [2]. Higher incidence and mortality are associated with populations from East Asia, Eastern Europe, and South America, while GC is less prevalent in Europe. Notably, the incidence of GC is rapidly increasing among younger individuals in developed countries, implying a shift in the risk factors associated with GC [3].

GC is a highly heterogeneous disease, with gastric adenocarcinoma being the most prevalent type [4]. Historically, gastric adenocarcinoma was histologically classified into intestinal-type or diffuse-type based on Lauren’s criteria [5], while, to date, the World Health Organization has recategorized GC into 4 subtypes: papillary, tubular, mucinous, and poorly cohesive. The incidence of diffuse-type GC is higher in females and younger individuals [6], whereas intestinal-type GC is more commonly associated with metaplasia and *Helicobacter pylori* infection [7,8]. Chronic inflammation in the gastric mucosa is a hallmark of GC development, and it is widely recognized that inflammation drives the pathogenic progression from chronic gastritis through stages of atrophic gastritis, intestinal metaplasia, dysplasia, to ultimately intestinal-type GC [9].

*H. pylori* infection is the most recognized risk factor of GC [10]. However, although *H. pylori* infects approximately 50% of the global population, less than 3% of the infected individuals develop GC [11], highlighting the contribution of additional factors to gastric tumorigenesis. Indeed, GC arises from a complex interplay of environmental and biological influences. For instance, clinical studies have reported the positive correlation between GC risk and diets rich in processed meats and N-nitroso compounds [12]. Concurrently, recent advances in microbial profiling have uncovered substantial shifts in the gastric microbiota during gastric tumorigenesis [13]. Given the crucial role of diet in shaping the composition and function of microbiota [14], interactions between diets and commensal microbes likely contribute to GC development. In this review, the impacts of different diets on gastric tumorigenesis are explored. The intricate interplays between diets and gastric microbiota in GC are also examined, with further evaluation of potential dietary interventions for GC prevention and management.

## Diets and GC

**Fig. 1.**
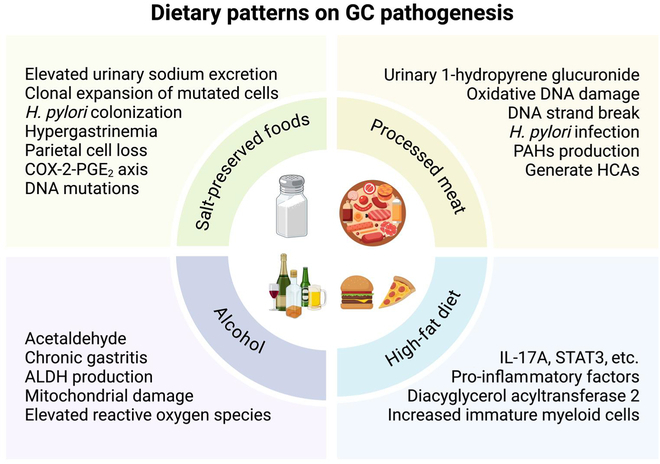
Dietary patterns and their pathogenic mechanisms in gastric cancer. Dietary patterns including salt-preserved foods, processed meat, alcohol, and high-fat diet influence gastric tumorigenesis through different mechanisms. (a) Excess dietary salts elevate urinary sodium excretion and promote *H. pylori* colonization and clonal expansion of mutated epithelial cells. Chronic salt exposure induces oxidative DNA damage by increasing reactive oxygen species, which synergize with *H. pylori* to trigger genomic instability. Excess salts also activate the COX-2-PGE_2_ axis, thereby promoting cellular proliferation and disrupting gastric epithelial homeostasis. (b) Carcinogenic compounds like HCAs and PAHs are generated in processed meat during cooking, which can induce mitochondrial dysfunction and cause double-strand breaks in DNA, resulting in mutations. (c) Alcohol is metabolized to generate acetaldehyde in the stomach, a metabolite known to disrupt gastric mucosal barrier and induce mitochdonrial damage with elevated reactive oxygen species. The accumulation of toxic aldehydes also exacerbates DNA damage and leads to chronic gastritis, while chronic inflammation promotes immature myeloid cell infiltration and subsequently gastric tumorigenesis. (d) Excess dietary fats can up-regulate DGAT2 activity, thereby increasing reactive oxygen species and STAT3 signaling. This pro-inflammatory milieu further enhances IL-17A production to promote angiogenesis and the recruitment of immature myeloid cells. Figure was created with BioRender.com.

Diet plays an important role in GC development, of which overconsumption of certain foods including salt-preserved foods, processed meat, and alcohol is associated with increased risk of GC. Individuals with excess body weight or obesity also have higher susceptibility to this malignancy. Herein, the influence of different dietary patterns on GC pathogenesis is examined, as summarized in Fig. 1.

### Salt-preserved foods

Excess consumption of high-salt foods is a well-documented risk factor for GC [15]. Rodent studies have demonstrated that excess dietary salts promote carcinogen-induced gastric tumorigenesis [16]. In humans, a meta-analysis in 2022 reported a marked positive association between high-salt intake and GC among 38 case–control studies [17], while GC mortality is also strongly linked to urinary sodium excretion level [18]. Mechanistically, high-salt diet directly damages the gastric mucosa, induces DNA mutations, and triggers hypergastrinemia, thereby driving parietal cell loss and gastric tumorigenesis in mice [19]. Excess salts further exacerbate chronic inflammation by stimulating the production of prostaglandin E_2_ (PGE_2_) and various pro-inflammatory cytokines (e.g., tumor necrosis factor-α, interferon-γ, interleukin [IL]-1β, and IL-6) in the gastric mucosa. Notably, high-salt treatment could amplify the cyclooxygenase-2 (COX-2)-PGE₂ pathway in mice with carcinogen (N-nitroso-N-methylurea [MNU])-induced GC, while COX-2 up-regulation then disrupts gastric epithelial homeostasis by increasing cellular proliferation and causing DNA damage and clonal expansion of mutated cells [20]. In addition, high-salt diet creates a favorable environment for *H. pylori* colonization, accelerating gastric atrophy and GC progression [21].

### Processed meat

Frequent intake of barbecued meat is strongly associated with elevated risk of GC [22], since prolonged cooking methods at high temperature, such as grilling or barbecuing, generate heterocyclic amines (HCAs)—a class of well-established mutagens and carcinogens. To date, 22 HCAs have been fully characterized in terms of their molecular structure [23,24]. In humans, HCA degradation begins with N-oxidation, primarily catalyzed by cytochrome P-450 enzymes, followed by enzymatic esterification by acetyltransferases or sulfotransferases, resulting in the formation of reactive nitrenium ions [25]. These reactive ions can create covalent bonds in DNA, particularly with guanine. DNA adducts can also be formed by HCAs without any enzymatic activation [26]. Once activated, HCA metabolites can induce oxidative DNA damage and cause double-strand breaks in DNA, resulting in mutations. Of note, it remains unclear whether HCAs have a combined or synergistic effect with *H. pylori* to induce DNA damage in gastric mucosal cells, which is a prerequisite for GC transformation. Furthermore, barbecued or grilled meats also induce the production of another carcinogen, polycyclic aromatic hydrocarbons (PAHs) [27]. PAHs can be metabolized into urinary 1-hydroxypyrene glucuronide, which is associated with increased risk of GC [28].

### Alcohol

Alcohol is one of the most well-established causes of cancer. A study in 2010 highlighted the link between alcohol consumption and increased risk of gastric adenocarcinoma [29]. A subsequent meta-analysis reported similar results, showing the positive correlation between the risk of GC and heavy alcohol drinking [30]. Apart from GC, early studies demonstrated that alcoholism also contributes to the development of precancerous chronic gastritis [31,32]. Mechanistically, alcohol drinking can increase the level of toxic metabolites in the stomach. In particular, alcohol dehydrogenase and catalase in the gastric mucosa can metabolize ethanol into acetaldehyde, which is then oxidized to acetate by aldehyde dehydrogenase (ALDH), a metabolite known to induce mitochondrial damage and elevate reactive oxygen species, thereby promoting gastric tumorigenesis and enhancing invasion of tumor cells [33,34].

### High-fat diet

Excess intake of dietary fats or Western diet is associated with increased risk of GC. In a preclinical study, intestinal metaplasia and gastric up-regulation of the pro-tumorigenic leptin signaling pathway were observed in mice fed with high-fat diet (HFD), suggesting a link between HFD-induced changes and the development of precancerous gastric lesions [35]. For GC, HFD-fed mice exhibited an accelerated progression of *Helicobacter*-induced gastric tumorigenesis, concomitant with increased trafficking of bone marrow-derived immature myeloid cells in gastric tissues and circulation [36]. HFD supplementation also elevates gastric levels of various pro-inflammatory factors such as IL-17A, granulocyte macrophage colony-stimulating factor, and phosphorylated signal transducer and activator of transcription 3 (STAT3) in *Helicobacter*-infected mice. These findings therefore imply that obesity plays a crucial role in inducing gastric inflammation and immunomodulation, potentially contributing to the progression of *Helicobacter*-induced GC. Moreover, a preclinical study in 2020 found that HFD promotes fat accumulation and peritoneal dissemination of GC in mice, of which peritoneum-derived adipocytes induce lipid accumulation and fatty acid oxidation in gastric tumor cells through up-regulating diacylglycerol acyltransferase 2 (DGAT2) transcription in a C/EBPα-dependent manner [37].

## Gastric Microbiota in Gastric Tumorigenesis

Human gastrointestinal tract harbors trillions of microbes to form the microbiota. Compared to the intestinal microbial community, microbes that reside in the stomach are much less studied and characterized. The gastric microbiota in humans is mainly composed of bacterial genera *Veillonella, Rothia, Prevotella, Streptococcus, Fusobacterium, Pasteurellaceae, Neisseria, Haemophilus, Actinomyces*, and *Porphyromonas* [38]*.* While the gastric microbiota is maintained at homeostasis under normal conditions, its composition and function are dysregulated throughout the progression of GC. Indeed, increasing studies have demonstrated the occurrence of microbial dysbiosis along gastric tumorigenesis. Several opportunistic pathobionts including *Streptococcus angionosus, Parvimonas micra, Dialister pneumosintes, Peptostreptococcus stomatis, Prevotella intermedia,* and *Fusobacterium nucleatum* are importantly enriched in gastric mucosal samples from patients with GC [39]. The gastric microbiota is also altered along the progression from gastritis, precancerous lesions, to GC. This includes enriched *Lactobacillus coleohominis* and Lachnospiraceae during gastric tumorigenesis, alongside with the depletion of *Porphyromonas*, *Neisseria*, and *Streptococcus sinensis* [40]. Other studies revealed a progressive shift in the gastric microbiota and highlighted the enrichment of oral or intestinal commensal microbes in the GC [41]. Moreover, microbial features can be utilized as biomarkers of GC. For example, a longitudinal prospective study reported 6 bacterial taxa at baseline that could predict the risk of future GC development, which include Comamonadaceae, *Moryella*, *Vibrio*, *Paludibacter*, *Agrobacterium*, and *Clostridium* [42].

Gastric microbial dysbiosis with enriched pathobionts aggravates GC development by dysregulating immune response and promoting inflammation, DNA damage, and epithelial–mesenchymal transition [43]. Increasing studies are investigating the functional and mechanistic role of GC-enriched bacteria in gastric tumorigenesis. For example, *S. anginosus* and *Streptococcus constellatus* are 2 oral pathogens markedly enriched in both gastric mucosa and stools of patients with precancerous chronic gastritis or early GC [44]. In particular, our recent study in 2024 identified that *S. anginosus* exerts its pro-tumorigenic effect by directly binding to the receptor Annexin A2 on gastric epithelial cells through its surface adhesin TMPC [45]. This host–bacteria interaction initiates multiple oncogenic signaling cascades, particularly the mitogen-activated protein kinase pathway, thereby promoting cell proliferation and inflammatio*n*. Another oral pathogen, *F. nucleatum*, is also associated with GC, of which enriched *F. nucleatum* could up-regulate exosome long noncoding RNA (HOX transcript antisense RNA) in gastric tumor cells, subsequently contributing to GC progression via the microRNA-885-3p/EphB2/PI3K/AKT pathway [46]. Moreover, an observational study in 2022 investigated the impacts of bile refluxing on the gastric microbiota and GC, reporting the marked enrichment of lipopolysaccharide (LPS)-producing bacteria (e.g., *Prevotella melaninogenica*, *Prevotella jejuni*, *Veillonella parvula*, and *Veillonella atypica*) in patients with bile reflux gastritis or GC [47]. The abundance of these LPS-producing bacteria is correlated with elevated conjugated bile acids, which together promotes GC by activating the pro-inflammatory IL-6/JAK1/STAT3 pathway. Meanwhile, STAT3 blockade could mitigate gastric tumorigenesis induced by bile refluxing in mice.

Given by their importance, numerous studies have assessed the potential of gastric microbes to serve as diagnostic biomarkers of advanced gastric adenocarcinoma. For instance, fecal enrichment of Enterobacteriaceae is associated with GC, accompanied by decreased microbial diversity [48]. By comparing GC to superficial gastritis, 5 GC-enriched bacteria taxa, *P. stomatis*, *S. anginosus*, *P. micra*, *Slackia exigua*, and *D. pneumosintes*, could accurately identify patients with GC with an area under the receiver operating characteristic curve of 0.82 [39]. Other studies reported enriched *Clostridium*, *Fusobacterium*, and *Lactobacillus* species in patients with GC, while these GC-enriched taxa particularly *Clostridium colicanis* and *F. nucleatum* possess robust diagnostic potential [49]. Nevertheless, further investigations are needed to have a clearer understanding on the mechanistic role of more gastric microbes in GC, prior to their applications in clinical diagnosis [50].

### Interplay between *H. pylori* and gastric microbiota in gastric tumorigenesis

*H. pylori* utilizes various mechanisms to favor its survival within the hostile acidic environment in the human stomach [51]. In infected individuals, *H. pylori* is the predominant microbial species in the gastric microbiota, accounting for 40% to 90% of microbial population in the stomach [52]. Given its overdominance, *H. pylori* importantly influence the overall composition of gastric microbiota in infected individuals, and it is as expected that *H. pylori* is negatively correlated with gastric microbial diversity [53–55]. *H. pylori-*infected individuals generally have a higher abundance of Proteobacteria due to the presence of *H. pylori*, and lower abundance of Actinobacteria, Bacteroidetes, and Firmicutes, compared to noninfected individuals [51,53]. An early study compared the gastric microbiota between *H. pylori*-infected individuals and noninfected individuals after excluding *H. pylori* [51]. Interestingly, the results revealed higher phylotype evenness and diversity in *H. pylori*-infected individuals, while no substantial difference was observed in microbial taxonomy and composition based on infection status [51]. Similarly, a preclinical study using a rhesus macaque model also reported that there is insignificant change of non-*Helicobacter* microbes before and after inoculation of *H. pylori* [56]. These findings therefore suggest that the gastric microbial community is relatively stable and unaffected by *H. pylori* infection. On the other hand, chronic infection of *H. pylori* could result in decreased acid secretion, potentially facilitating the growth and colonization of microbes that are not indigenous to the stomach, resulting in gastric microbial dysbiosis [57]. In addition, *H. pylori*-induced gastric microbiota alterations appear to be reversible, as *H. pylori* eradication leads to an increase in microbial diversity in the human stomach [58,59]. Notably, this observation could be alternatively explained by *H. pylori* being part of the “normal” gastric microbiota, whereas *H. pylori* eradication may, in fact, disturb microbial homeostasis in the stomach.

Multiple studies have profiled the progressive depletion of *H. pylori* in gastric tumorigenesis, of which *H. pylori* may even become undetectable in GC [39]. As the gastric microbiota between GC and precancerous patients is markedly different, these findings imply the potential pro-tumorigenic interactions between *H. pylori* and other gastric microbes. Hence, eliminating *H. pylori* may, in turn, avoid these microbial interplays, thereby suppressing gastric tumorigenesis. Indeed, a study found that *H. pylori* eradication could protect against GC by restoring a dysbiotic gastric microbiota [60]. Another study compared the gastric mucosal microbiota between individuals with and without atrophy development 1 year after *H. pylori* eradication [58]. In individuals without atrophy, more beneficial bacteria including *Acinetobacter, Faecalibacterium, Kaistobacter, Blautia, Caulobacter,* and *Brevundimonas* were observed, in contrast to the enrichment of opportunistic pathogen (e.g., *Granulicatella, Streptococcus, Rothia*, and *Leptotrichia*) in individuals with emerged atrophy after *H. pylori* eradication. Subsequent functional analysis identified an increase in energy generation and stress adaptation in the gastric microbiota of gastritis patients. Nonetheless, to date, the mechanistic role of gastric microbes in GC remains largely elusive. Further studies that utilize more advanced technology such as spatial profiling are suggested, which may provide new insights into the landscape of gastric microbiota in gastric tumorigenesis.

### Metabolite alterations in GC

Apart from gastric microbes, gastric tumorigenesis is also profoundly shaped by various metabolites, which interact with host cells to drive inflammation, DNA damage, and immune evasion (Table). For example, secondary bile acids (e.g., deoxycholic acid and lithocholic acid), chiefly produced by *Clostridium* and *Bacteroides* species, activate the nuclear factor kappa B (NF-κB)/STAT3 signaling pathway to promote DNA damage, cell proliferation, and inflammation in the gastric mucosa [13]. These effects are further potentiated in a hypochlorhydria environment, where oral anaerobes such as *Prevotella* and *Veillonella* convert dietary amines into toxic N-nitroso compounds to induce DNA alkylation and mutagenesis [61]. Hydrogen sulfide generated by *Desulfovibrio* could also activate NF-κB signaling to inhibit apoptosis and promote angiogenesis [13]. Moreover, *Klebsiella*-derived acetaldehyde forms mutagenic DNA adducts [61], while *Enterococcus* produces methylglyoxal to induce oxidative stress through advanced glycation end-products [13]. In contrast, pentadecanoic acid from beneficial *Akkermansia muciniphila* enhances oxaliplatin sensitivity by targeting glycolytic enzymes (HK2/PGK1), thus offering a novel therapeutic avenue [62].

**Table.** Metabolite alterations in gastric cancer

Metabolite	Microbial producer	Mechanism	Sample type	Ref.
Secondary bile acids	*Clostridium*, *Bacteroides*	DCA/LCA activates NF-κB/STAT3 and induces DNA damage and cell proliferation	Human gastric mucosa	[13]
N-nitroso compounds	Oral anaerobes (e.g., *Prevotella*, *Veillonella*)	Nitrosation of amines in low-pH stomach, DNA alkylation	Human gastric juice	[61]
Putrescine	*Bacteroides*, *Proteus*	Inhibit histone deacetylases → epigenetic silence of tumor suppressors	Human gastric tumors	[64]
SAM	*Streptococcus anginosus*	Compete with host cells for arginine → CD8^+^ T-cell exhaustion	Human gastric mucosa	[65]
Lipoteichoic acid	*Lactobacillus*	Activate toll-like receptor 2/4 → IL-6/STAT3 signaling → chronic inflammation	Mouse gastric tissues	[13]
Hydrogen sulfide	*Desulfovibrio*	Inhibit apoptosis via NF-κB activation and promote angiogenesis	Human gastric biopsies	[13]
Acetaldehyde	*Klebsiella*	DNA adduct formation (e.g., N2-ethylidene-dGuo) → mutation in tumor protein 53	Human gastric washes	[61]
Methylglyoxal	*Enterococcus*	Advanced glycation end-products → ROS generation and oxidative DNA damage	Mouse gastric mucosa	[13]
Bilirubin derivatives	*Bilophila*	Disrupt mitochondrial function via ROS → apoptosis resistance	Human gastric tissues	[13]
Peptidoglycan	*Peptostreptococcus*	Activate NOD1/2 → NF-κB-driven chronic gastritis	Human gastric biopsies	[64]
Butyrate	*Faecalibacterium*, *Roseburia*, *Akkermansia*	Inhibit histone deacetylases and suppress PD-L1/IL-10 in tumor-associated macrophages; modulate CD8^+^ T cell immunity via GPR109A	Human gastric tumors	[66,67]
Pentadecanoic acid (C15:0)	*Akkermansia muciniphila*	Modulate glycolysis and enhance oxaliplatin sensitivity by targeting HK2/PGK1	Mouse gastric xenografts	[62]
Methylarginines	*Enterococcus*, *Citrobacter*	Inhibit nitric oxide synthase and promote angiogenesis and metastasis	Human gastric tumors	[63]
Lipid peroxidation products	*Helicobacter pylori*	Induce ferroptosis resistance via GPX4 up-regulation	Human gastric tumors	[63]

DCA, deoxycholic acid; LCA, lithocholic acid; ROS, reactive oxygen species

Metabolomic profiling of human gastric tissues, mucosal biopsies, and gastric juices reveals various key metabolites in different tumor locations of GC. In particular, proximal gastric tumors are associated with elevated methylarginines and lipid peroxides, which links to angiogenesis and metastasis, whereas distal tumors have higher levels of β-alanine and DNA repair inhibition [63]. Consistent with human findings, *Lactobacillus*-derived lipoteichoic acid synergizes with *H. pylori* to amplify chronic inflammation via toll-like receptor 2/4 signaling in mice [13]. Intratumoral metabolites can also modulate the tumor microenvironment. For instance, putrescine, synthesized by *Bacteroides* and *Proteus* species, inhibits histone deacetylases to induce epigenetic silencing of tumor suppressors [64]. *S. anginosus* secretes the metabolite SAM to cause metabolic disruption and compete with host cells for arginine, thereby triggering CD8^+^ T-cell exhaustion and impairing antitumor immunity [65]. On the other hand, butyrate (derived from *Faecalibacterium*, *Roseburia*, and *Akkermansia*) could modulate antitumor immunity by down-regulating IL-10 and programmed death ligand 1 (PD-L1) in tumor-associated macrophages, while enhancing CD8^+^ T-cell cytotoxicity [66,67]. Clinically, these metabolites may potentially serve as diagnostic biomarkers and therapeutic targets of GC. Future studies should therefore prioritize longitudinal investigation of host–microbe and metabolite interactions to develop microbiota-targeting therapeutic strategies, thereby inhibiting gastric tumorigenesis.

### Gut microbiota and metabolites in GC immunomodulation

Besides tumorigenesis, recent findings have highlighted the association of gut microbiota with immunomodulation in patients with GC. A clinical and murine study in 2024 reported the elevation of immunosuppressive markers PD-L1 and IL-10 in immune cells (e.g., macrophages and dendritic cells) and tumor tissues from patients with advanced GC, compared to healthy individuals [66]. Microbiota analysis revealed decreased microbial diversity and reduced butyrate-producing bacteria such as *Faecalibacterium* and *Bifidobacterium* in GC, while such depletion is correlated with disease progression. Of note, supplementation of microbe-derived butyrate substantially suppresses PD-L1 and IL-10 expression as well as tumor growth in mouse xenografts, concomitant with down-regulation of immunosuppressive pathways (e.g., NF-κB and STAT3) and pro-tumorigenic factors (e.g., VEGF and GDF-15) [66]. Consistently, another murine study also reported that exogenous butyrate treatment importantly inhibits gastric tumorigenesis in carcinogen (MNU)-treated *H. pylori*-infected mice, yet the antitumor effects of butyrate are abolished after knockout of G protein-coupled receptor 109A (GPR109A) [67]. Moreover, through fecal microbiota transplantation, mice receiving stools from human patients with GC have lower butyrate level and faster tumor growth than mice transplanted with stools from healthy individuals, hence confirming the protective role of butyrate in GC. Mechanistically, butyrate boosts the tumor-killing capacity of CD8^+^ T cells and Claudin 18.2-targeting CAR-T cells through the GPR109A-homologous domain protein homologous box (HOPX) pathway, thereby restoring antitumor immunity and thus suppressing gastric tumorigenesis [67].

By contrast, certain bacteria and their metabolites can promote GC. Through multi-omics analysis of GC tumor tissues, *S. anginosus* was identified in a marked correlation with worse prognosis [65]. In mice with carcinogen (MNU)-induced GC, *S. anginosus* not only promotes gastric tumorigenesis as reported in another study [45], but also suppresses the differentiation and infiltration of cytotoxic CD8^+^ T cells. Further metabolomic profiling and spatial imaging revealed that *S. anginosus* could metabolize arginine into ornithine, which is known to induce immune evasion and tumor progression [68]. These findings therefore designate *S. anginosus* as a pivotal pro-tumorigenic bacteria, emphasizing its potential as both a diagnosing biomarker and therapeutic target of GC [65]. Indeed, a multicenter observational study involving 1,043 Chinese patients reported that fecal *S. anginosus* plus *S. constellatus* could accurately distinguish patients with different stages of GC with superior sensitivity and specificity [44]. Nonetheless, while the application of microbial taxa and metabolites as diagnostic biomarkers for different cancers is widely reported [69], there is currently a lack of large-scale multi-population study of microbial biomarkers for GC. In addition, microbes are also associated with treatment response in GC. A prospective clinical trial in 2025 reported marked alterations in microbiota composition and function after immune checkpoint blockade [70]. For example, *F. nucleatum* is importantly enriched 8 weeks after treatment as compared to baseline. Meanwhile, higher baseline abundance of *Faecalibacterium prausnitzii* is correlated with better overall and progression-free survival, highlighting its potential as a predictive biomarker of immunotherapy response in patients with GC.

## Diets and Gastric Microbiota in GC

**Fig. 2.**
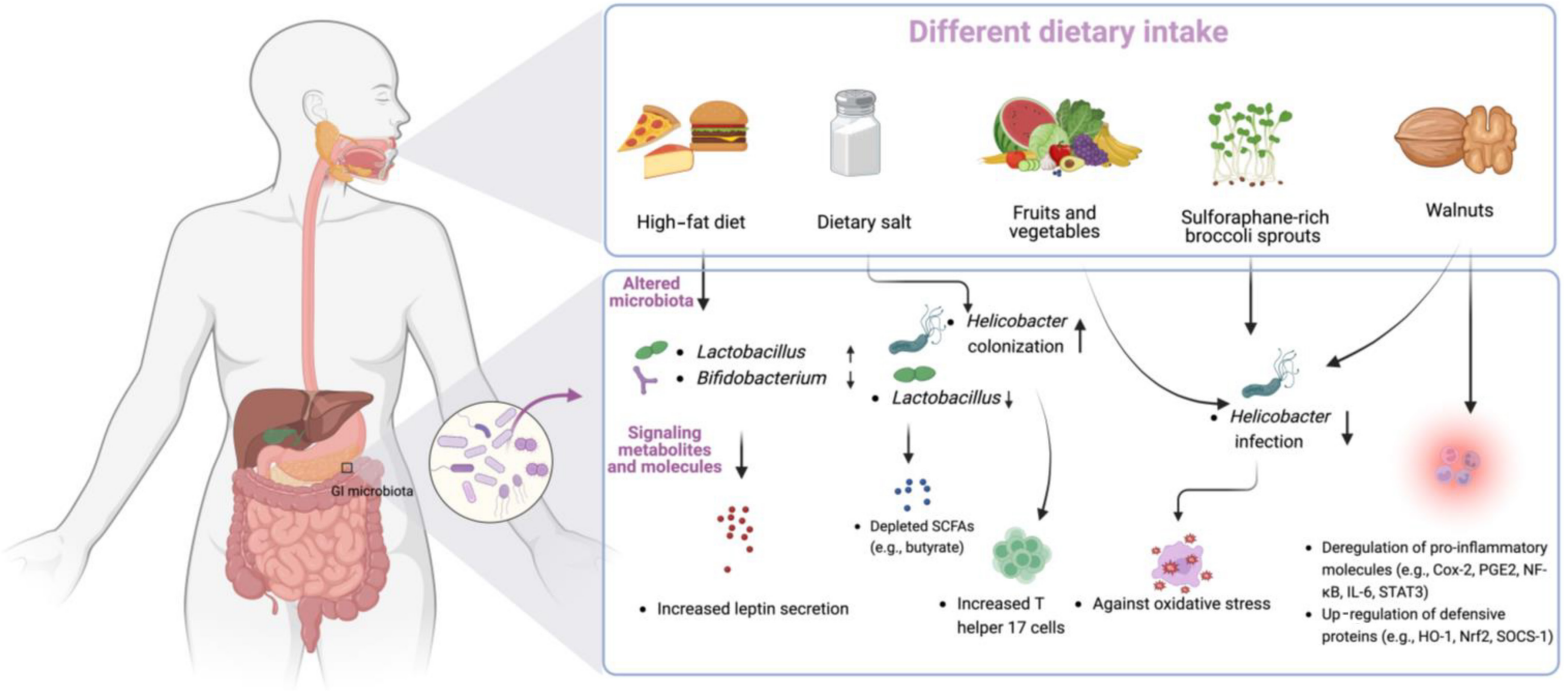
Interplays between diets and gastric microbiota in gastric cancer. Human gastric microbiota is heavily influenced by diets, whereas different diets have distinct effects on gastric tumorigenesis. High-fat diet induces gastric microbial dysbiosis with increased *Lactobacillus* and decreased *Bifidobacterium*, and promotes leptin secretion to activate pro-inflammatory pathways (e.g., COX-2/PGE_2_/NF- κ B). Excess dietary salts promote *H. pylori* colonization and deplete short-chain fatty acids (SCFAs) (e.g., butyrate), thereby disrupting the integrity of gastric mucosal barrier integrity and inducing IL-6/STAT3-mediated inflammation. On the other hand, fruits, vegetables (e.g., sulforaphane-rich broccoli sprouts), and walnuts can up-regulate the HO-1/NRF2 antioxidant pathway, while suppressing leptin production and pro-inflammatory factors (e.g., COX-2, PGE_2_, and STAT3), as well as *H. pylori* colonization. These features collectively facilitate the inhibition of gastric tumorigenesis. Figure was created with BioRender.com.

Human microbiota is heavily influenced by environmental factors, and among them, diet is undoubtedly one of the most critical elements that shapes the microbial communities and their functions [71]. While many studies have revealed the correlation between gut microbes and diets [72–74], there are much fewer investigations on how diets affect the gastric microbiota. In this section, the interplays between diets and gastric microbiota in GC development are elucidated (Fig. 2).

### Diet alters gastric microbiota

HFD is known to change the composition and function of gut microbiota [75–78], while recent evidence also reported its impact on gastric microbes. In a murine study, the gastric mucosal microbiota is markedly altered by HFD, with enriched Firmicutes and Proteobacteria, as well as depleted Bacteroidetes and Verrucomicrobia [79]*.* In particular, a remarkable reduction of beneficial bacteria, especially *A. muciniphila*, was observed in the stomach of HFD-fed mice. HFD supplementation also induces gastric microbial dysbiosis with increased *Lactobacillus* and decreased *Bifidobacterium* abundance in mice, leading to intestinal metaplasia and elevated leptin secretion. Moreover, unlike HFD-fed conventional mice, gastric microbiota dysbiosis and intestinal metaplasia were not observed in HFD-fed mice with leptin receptor knockout, indicating that HFD activates leptin signaling to modulate the gastric microbiota and promote gastric tumorigenesis [35]. On the other hand, leptin signaling could be inhibited by suppressor of cytokine signaling 3 (SOCO3), of which mice with SOCS3 conditional knockout in gastrointestinal epithelial cells develop gastric tumors that resemble human intestinal-type GC [80]. SOCO3 knockout leads to hyperplasia of gastric mucosa by increasing leptin production and causing hyperactivation of STAT3 signaling, which, in turn, implies the protective role of SOCO3 against GC.

As aforementioned, excess intake of dietary salts is positively associated with GC, while it can also alter the gastric microbiota to contribute gastric tumorigenesis. A preclinical study showed that the abundance of *Lactobacillus* decreases in mice fed with high-salt diet, concomitant with gastritis and increased pro-inflammatory T helper 17 cells [81]. Functional analysis revealed that high-salt diet suppresses the ability of gastric bacteria to metabolize polysaccharides and vitamins. Excess intake of sodium chloride also promotes the colonization of *H. pylori* in the stomach, which, in turn, exacerbates gastritis development and eventually progression to GC [19,81]. In addition, high-salt diet could exacerbate colitis in mice by depleting *Lactobacillus* and butyrate production, whereas these effects were not observed in germ-free mice, thus highlighting the role of microbiota to mediate the pro-inflammatory effect of dietary salts [82].

### Dietary intervention against GC

Given the crucial roles of diets in GC, it is feasible to apply dietary intervention against this malignancy. Indeed, diets rich in fruits and vegetables, and low in processed meat and salty foods, can importantly lower the risk of GC [83]. Gastric tumorigenesis can also be suppressed by diets rich in vitamin C and low salt content [12]. An early study reported that daily intake of sulforaphane-rich broccoli sprouts for 2 months reduces *H. pylori* colonization and improves sequelae in *H. pylori*-infected mice and humans, through enhancing chemoprotection against infection-induced oxidative stress in the gastric mucosa [84]. A preclinical study in 2021 showed that walnuts can be nutritional intervention to prevent *H. pylori*-associated GC, of which dietary intake of walnuts for 36 weeks suppresses gastric tumorigenesis in mice by down-regulating pro-inflammatory (e.g., COX-2, PGE2, NF-κB, IL-6, and STAT3) and proliferative factors (e.g., Ki-67 and PCNA), while up-regulating defensive proteins (e.g., HO-1, NRF2, and SOCS-1) [85]. In addition, enzymes involved in alcohol metabolism, particularly ALDH, also play a crucial role in tumorigenesis, progression, and treatment of various cancers, indicating their potential as therapeutic targets of GC [86]. Collectively, these preclinical findings have demonstrated the promising capacity of dietary interventions, offering anticipation for their clinical applications against GC.

## Current Limitations and Future Perspectives

It has been decades since the discovery of *H. pylori* and its pathogenic role in GC development. With the advance in microbial profiling technology, commensal microbes in the human stomach have received more attention and increasing studies have illustrated the marked associations of gastric microbiota with gastric tumorigenesis. However, a major issue of current studies is the lack of comparability, due to the disparity in sample processing, sequencing platform, and analytical methods across studies, not to mention the large variation of microbial communities among individuals [87]. Current studies also heavily focus on profiling the alterations of gastric microbiota under disease conditions, without mechanistic investigation on how gastric microbes contribute to gastric tumorigenesis. Moreover, many recent studies have reported the crucial involvement of nonbacterial microbes particularly viruses and fungi in the development and progression of other cancers [87,88]. Meanwhile, the role of gastric viruses and fungi in GC remains massively elusive, which might be explained by their extremely low abundance in human stomach. Taken together, compared to the intestinal microbiota, research on many fields of the gastric microbiota is still in the early stage. More in-depth investigations are required to fill the knowledge gaps of gastric microbes beyond *H. pylori* in gastric tumorigenesis.

**Fig. 3.**
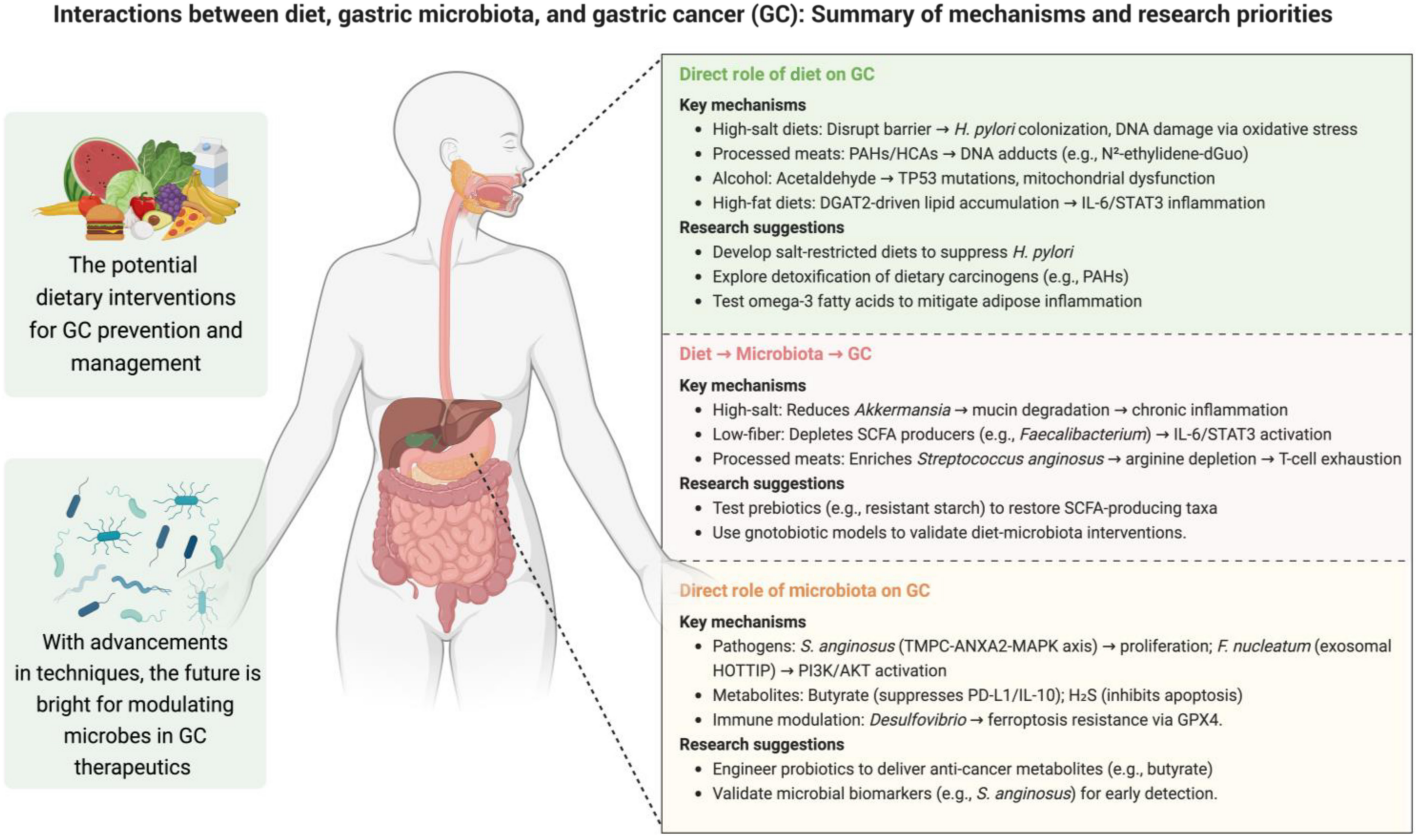
Summary of mechanisms and research priorities. An overview of the crosstalk and underlying mechanisms between diets and the gastric microbiota in GC. Certain diets (e.g., high-salt diet, processed meat, alcohol, and HFD) directly influence gastric tumorigenesis or induce changes in the gastric microbiota to contribute to GC development. Meanwhile, apart from *H. pylori*, a dysbiotic gastric microbiota with enriched pathogenic bacteria (e.g., *S. anginosus*) and their metabolites can also promote gastric tumorigenesis. Given their critical roles, future studies should emphasize actionable research priorities for prevention and therapy against GC. These priorities include tailored dietary interventions such as salt-restricted diets and prebiotics (e.g., resistant starch) to promote the growth of beneficial commensal bacteria, engineered probiotics to restore the gastric microbiota, and microbial biomarkers (e.g., *S. anginosus*) for early detection of GC. Figure was created with BioRender.com.

Evidence from epidemiological, preclinical, and clinical studies clearly supports the notion that diet plays a crucial role in the development of GC. On the other hand, dietary intervention can reshape and modulate the gastric microbiota, hence showing promising potential as a prophylactic strategy to inhibit gastric tumorigenesis. Based on current findings, a balanced diet rich in fruits and vegetables is highly recommended as a preventive measure of GC. Nevertheless, there are still numerous unsolved issues on utilizing dietary intervention to protect against GC, mostly owing to the unclear role and function of gastric microbes during gastric tumorigenesis. Another critical obstacle is to design diets that can specifically eliminate pathobionts without affecting beneficial commensals. The role of dietary factors in a disease is complex and multifactorial. For instance, while a specific dietary component is associated with increased risk of cancer (e.g., red meat), its pro-tumorigenic effect is lost when combined with other healthy diets (e.g., fruits and vegetables). In this case, a diet that includes red meat might be acceptable if other nutritional factors are present. To date, observational studies with documented dietary history are notoriously lacking, and future studies should be more cautious when considering the impacts of various specific diets on GC. Clinical longitudinal studies are also necessary to assess the efficacy, duration, and safety of dietary interventions to prevent GC development. In summary, future research should prioritize the development of tailored dietary interventions (e.g., sodium restriction and omega-3 supplementation), microbiota-targeting strategies (e.g., precision prebiotics and engineered probiotics), and microbial biomarkers (e.g., screening of GC-enriched *S. anginosus*) (Fig. 3). This urgency underscores the need to harness precision nutrition and microbiota-modulating therapies to interrupt oncogenic cascades, advocating for collaborative multidisciplinary frameworks in GC prevention and clinical care. Moreover, advancing the understanding of gastric microbiota may further pave the way for innovative dietary approach to halt gastric tumorigenesis.
